# Where should Momma go? Current nursing home performance measurement strategies and a less ambitious approach

**DOI:** 10.1186/1472-6963-7-93

**Published:** 2007-06-25

**Authors:** Charles D Phillips, Catherine Hawes, Trudy Lieberman, Mary Jane Koren

**Affiliations:** 1 Program on Aging and Long-term Care, Department of Health Policy and Management, School of Rural Public Health, Texas A&M University System Health Science Center, College Station, Texas, 77843, USA; 2 Center for Consumer Health Choices, Consumers Union, 101 Truman Avenue, Yonkers, NY 10703, USA; 3 Commonwealth Fund, 1 East 75th Street, New York, NY 10021, USA

## Abstract

**Background:**

Nursing home performance measurement systems are practically ubiquitous. The vast majority of these systems aspire to rank order all nursing homes based on quantitative measures of quality. However, the ability of such systems to identify homes differing in quality is hampered by the multidimensional nature of nursing homes and their residents. As a result, the authors doubt the ability of many nursing home performance systems to truly help consumers differentiate among homes providing different levels of quality. We also argue that, for consumers, performance measurement models are better at identifying problem facilities than potentially good homes.

**Discussion:**

In response to these concerns we present a proposal for a less ambitious approach to nursing home performance measurement than previously used. We believe consumers can make better informed choice using a simpler system designed to pinpoint poor-quality nursing homes, rather than one designed to rank hundreds of facilities based on differences in quality-of-care indicators that are of questionable importance. The suggested performance model is based on five principles used in the development of the Consumers Union 2006 Nursing Home Quality Monitor.

**Summary:**

We can best serve policy-makers and consumers by eschewing nursing home reporting systems that present information about all the facilities in a city, a state, or the nation on a website or in a report. We argue for greater modesty in our efforts and a focus on identifying only the potentially poorest or best homes. In the end, however, it is important to remember that information from any performance measurement website or report is no substitute for multiple visits to a home at different times of the day to personally assess quality.

## Background

Almost all nursing homes, whether they provide good, bad, or indifferent quality of care, engage in a wide range of care activities for a relatively diverse set of residents. A few homes do most of these activities well, and a few homes do most of these activities badly. Many more operate in a world defined by variations in performance where one sees a mix of the good and the bad, or simply observes shifts among levels of mediocrity [1].

Given the diversity of nursing homes and their varying abilities to provide appropriate service, what advice can researchers offer policy-makers or consumers interested in differentiating among homes based on quality of care? In the past, health services researchers have explored various means of reporting nursing home performance that focused on all the homes in an entire nation, state, or area. We argue here that we in the research community may, in essence, have promised more than we can truly deliver. We believe that currently strategies based on quantitative quality indicators, no matter how successfully implemented, are seriously flawed. These flaws are rooted in the inherent characteristics of nursing homes and nursing home residents and stymie researchers in their attempts to differentiate between nursing homes providing different levels of quality of care. These barriers to successful performance measurement include the:

• complex nature of quality in nursing homes [2],

• diversity of the nursing home population [3],

• lack of knowledge about how homes, as organizations, generate quality [4],

• validity of comparisons among homes using current quality indicators [5].

These factors in combination create fundamental problems in our ability to provide consumers with meaningful evaluations of nursing home performance [6].

With such a litany of obstacles facing current nursing home performance measurement systems, one might suggest simply scraping the entire effort. To the contrary, we believe such systems can provide good information to both consumers and policy makers. We simply argue that these performance measurement systems can probably be used relatively well for telling consumers "where Momma shouldn't go." These systems may even be able to provide options to be considered for "where Momma should go." It is after developing these two relatively small categories of facilities that we have no good response to the specific question of whether Momma should go to "Home Z," if it falls outside the bounds of those identified as the "good" or the "bad".

For better or for worse, nursing home performance measurement systems have become quite popular over the last few years [7]. Many of these systems attempt to provide consumers information based on percentile rankings, three level rankings (e.g., above average, average, below average) or five level ranking systems for homes on a variety of dimensions to answer the question of whether Momma should go to "Home Z".

Unfortunately, we believe such rankings are of questionable validity. However, the problems inherent in current nursing home performance measurement could be overcome, in part, by shifting the focus making pronouncements about all homes to identifying the best and the worst homes. Before exploring our preferred option for these systems, we discuss in some detail the barriers we believe prevent the current systems from providing useful information.

## Potential problems in nursing home performance measurement

### The multi-dimensionality of quality

**Figure 1:**
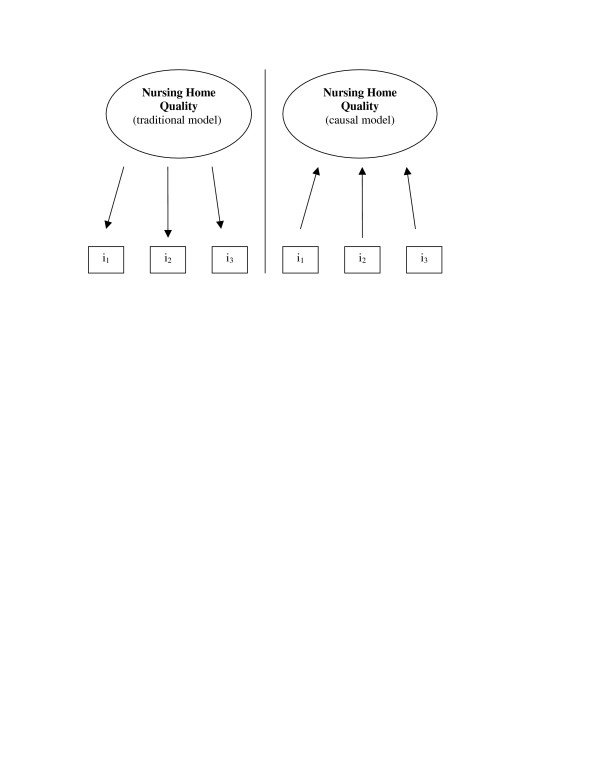
Traditional and causal measurement models for nursing home quality. Source: adapted from Bollen & Lennox, 1991.

Our initial problem arises because quality is multidimensional, making it difficult to summarize the quality of care in individual nursing home facilities. In most measurement situations, we operate within the confines of a traditional measurement model. We deal with some unmeasured entity (quality) represented by relatively highly correlated indicators. In the world of nursing home care our situation is much more analogous to what Bollen and Lennox [8] identify as a *causal model of measurement*. Changes in quality do not so much move correlated indicators of quality as changes in marginally, or uncorrelated, indicators move the larger unmeasured construct of quality (see Figure 1). A home may improve skin care, theoretically increasing the value of our unmeasured construct of quality, but that change will have no effect on that home's use of feeding tubes, another reasonable and appropriate indicator for that larger unmeasured construct.

As an illustration, in one study, the falls quality indicator from the Minimum Data Set for Nursing Home Resident Assessment and Care Screening (MDS) was correlated with 24 other quality indicators (QIs). The average correlation of these 24 indicators with the falls indicator was 0.06, with seven of the correlations being negative [2]. John Hirdes and colleagues [1] used an interesting visual display that illustrates the same lack of high correlations even more vividly. They presented QI percentile ranking for multiple QIs for a single home in a radar graph (analogous to a circular bar graph). Ideally, the multiple rankings would form a perfect circle or, at least, an oval. Instead, far more often, the result was an uneven field of peak and valleys, giving one little hope of high correlations among the quality indicators for a single facility. Were such high correlations present, it would be much easier to think of how one might develop some single scale based on diverse quality indicators that would allow one to talk about overall quality of care in homes.

At this point one may wonder whether quality measurement at the home level is a useful endeavor. If we can't find a set of highly correlated indicators, then some might argue that the logic of scientific inquiry suggests that we look for a less general conceptualization, or a series of dimensions of quality, that might be composed of more highly correlated items. Possibly, as some suggest, deeper inquiry into the dimensionality of quality will provide assistance [5] as might being more conceptual in our thinking about quality [9].

This might, in fact, be a useful exercise in a largely academic inquiry into quality. In the applied world of nursing home operations, however, these less than ideal, poorly correlated indicators; the care problems they may represent; and the residents who may be at risk in each care area all appear in a single home. Residents can't get their skin care from one home because of its superior performance on this indicator and get their incontinence care from a different home because of its performance on this quality indicator. Residents receive all their care in a single home.

But, it is not only residents and potential residents who need information on facility-level quality. Regulators may wish to develop monitoring systems that involve more serious scrutiny for more problematic providers. Policy-makers are also becoming more interested in payment-for-performance, which will demand an assessment of facility-level quality [10]. In all of these instances, entire homes must be discussed. However, what remains unclear, due in part to the multi-dimensionality of quality, is how much one can or should say about individual homes?

### The multi-dimensionality of the nursing home population

One potential solution for concerns about the multi-dimensionality of quality, at least as it concerns consumers, has been to provide a wide array of information [7]. For homes across the country, consumers can now acquire online data on deficiencies in multiple surveys, staffing levels, financial data, and quality indicators. What many consider the best of these sites tells a visitor whether the information indicates the home is average, above average, or below average on some indicator. But, is average care good or bad? How do consumers integrate such data on multiple, potentially uncorrelated, indicators?

Even if these systems are used and consumers can integrate the disparate pieces of information they received about a home, one faces a second problem, which is the multidimensionality of the nursing home population. Part of the difficultly in disseminating quality information is dealing with the array of very diverse residents that populate nursing homes. The NH Compare site operated by CMS differentiates between quality indicators for post-acute and for long-stay residents. This is an important step in such differentiation, but it glosses over much of the important variation among residents.

If a resident is bedfast and incontinent, family members, surveyors, and ombudsmen should care tremendously about skin and continence care. If the resident suffers from dementia but is physically active, they should care much more about safety, restraint policy, and activities. Robert Kane [3] has identified at least five different groups of residents who come into nursing homes. These groups include residents recovering from an acute episode and who are likely to return home, residents who are terminally ill, residents who are cognitively impaired, resident who are cognitively intact but suffer from physical challenges, and residents in vegetative states. All of these residents have different needs and different dimensions of quality will vary in their importance across these groups.

Discussing quality becomes even more complicated when we recognize that even within these five groups, residents themselves may entertain varying definitions of quality. As Barbara Bowers and her colleagues [11] indicate, some residents define quality in terms of service, others in terms of attention to comfort, while others define quality in terms of the nature of their interpersonal relationships with staff. Yet, our current level of sophistication in the targeting of quality indicators to special populations goes not deeper than recognizing the distinction between short-stay and long-stay residents.

### Home-related variation in quality indicators

Another issue demanding much of our attention is resident outcomes. Unfortunately we have little idea just how much of the variation in resident outcomes is driven by a home's performance. A recent analysis of quality of life indicators [12] implies that such measures can distinguish among homes. However, the analyses indicated that that home characteristics such as size or ownership, explained less then 10% of the variation in quality of life. The analyses of MDS quality indicators considered for inclusion in the CMS NH Compare website required that nursing home characteristics explain at least 21% (r = .45) of the variation in a quality indicator [13] to meet their criteria for validity. It is a less than heartening situation when home characteristics explain just over one-fifth of the variation in an outcome or quality indicator, and we consider that indicator a "valid" measure of homes' performance.

As a group, researchers in nursing home quality seem to have largely avoided dealing with a critical issue. *How much variation in a quality indicator must home performance explain in order for the research community to consider the indicator useful*? While there is no real answer to such a question, since at this point it is fundamentally a matter of "taste," one can ask a similar question that is much more answerable. Which of our array of potential quality indicators are most affected by home performance? If 90% of the variation in Quality Indicator #1 is determined by factors outside the control of a home and only 35% of the variation in Quality Indicator #2 is determined by factors outside the control of the home, then Quality Indicator #2 may be more useful as an indicator of home performance. Unfortunately, this is not a question that is being addressed by the research or policy communities.

### Risk adjustment and the validity of quality indicators

The face, content, and construct validity of the measures developed for nursing homes are relatively rarely points of debate, though a great deal of effort has been expended to ascertain how valid specific measures are when compared to direct observations of care [14-17]. In fact, much of this work has shown that current indicators poorly reflect observed care in homes. However, the validity that might be gained by substituting much more costly observations of care for the current indicators is not clear at this time.

The most often raised issue concerning the validity of the quality measures seems to concern the degree to which one can successfully risk adjust these measures [6,5]. However, prior to the successful risk adjustment, one needs some clearer sense about the degree of covariance between homes and residents before beginning to understand how important risk adjustment may be. If only 21% of the variation in an indicator can be explained by home characteristics, then how much of the residual can be driven by the covariation of home and individual characteristics? How high must that covariance be in order to demand risk-adjustment?

Even when we make the decision to risk-adjust quality indicators, the task is far from easy. In essence, we are like Goldilocks with only two bears. For everything Goldilocks tried when she was engaged in breaking and entering at the Bear residence, whatever was meant for the one bear was too much, while whatever was meant for another bear was not enough. Only when she tried the bed or porridge of the third bear, was it "just right." When we risk adjust, we must consciously choose whether to go with Bear #1 and "over-adjust" or with Bear #2 and "under-adjust." Unfortunately, there is no third bear conveniently available to offer us the risk-adjustment model that is "just right."

Fortunately, our adjustments will rarely be so powerful that they make the truly good homes look bad or the truly bad homes look good. However, when we over-adjust, we run the risk of making bad homes look mediocre, and under-adjusting may make good homes look mediocre. When we compare the cost of erring in either direction, it seems that the most reasonable course is to consciously under-adjust. Given the option, we provide better service to consumers and other stakeholders (except those facilities that are mediocre and identified as bad) by failing to give a few mediocre homes their due than to make anyone think that a bad home is just mediocre.

## Nursing home performance measurement: a less ambitious approach

Despite the problems and unanswered questions noted above, we do not want "the perfect to be the enemy of the good" in the field of nursing home performance measurement. Researchers can deal only partially with these issues and still provide other researchers, policy-makers, and consumers with useful information.

Over the years, Consumer Reports has published a series of Nursing Home Watch Lists [18,19]. These Watch Lists identified the "worst 10%" of nursing homes in each state, based solely on the review of each home's record over its three to four most recent annual certification/licensure surveys by the state survey agency.

However, in the development of the most recent Consumers Union 2006 Nursing Home Quality Monitor, a completely new strategy was used. The strategy was based on a series of five simple principles.

1. A home's relative values on multiple dimensions of quality should be considered.

2. The home's performance over time should be included in the analysis.

3. How a home fared on each dimension of quality over time should be aggregated into a single summary statement about a home.

4. The results should be used only to identify facilities that score very badly or very well on multiple dimensions of quality.

5. One should place more confidence in the possibility that a home may provide poor care than that a home may provide high quality care.

These principles are discussed below in the context of the Consumers Union 2006 Nursing Home Quality Monitor [20]. The discussions of the process for developing each indicator are not highly detailed. Instead, the discussions are an attempt to illustrate the principle under discussion. A full discussion of each step in the development of the 2006 Nursing Home Quality Monitor is available elsewhere [4].

This approach to nursing home performance measurement is less ambitious in two senses. First, it does not attempt to rate all homes. It focuses on what seem to be the poorest and best performers. Second, the approach is also somewhat lacking in ambition in that it does not attempt to remedy the problems noted above. Neither do we suggest entirely new strategies for gathering quality data, nor do we enumerate entirely new approaches to thinking about home performance. Our approach simply takes what is currently available for performance measurement, accepts the existence of problems, and attempts to devise a rating model that we believe minimizes the impact of these problems.

### Using multiple dimensions of quality

As noted earlier, nursing home quality is clearly multi-dimensional. So, in developing performance measurement systems for nursing homes, one must use multiple dimensions of quality. In developing performance measurement algorithms for the Consumers Union 2006 Nursing Home Quality Monitor, we considered three basic dimensions of quality: nurse staffing levels, the number and type of survey deficiencies, and a subset of CMS quality indicator scores from the Nursing Home Compare website.

Homes were rated separately on each dimension. Each dimension had its own scoring process and within each state those homes that scored in the poorest 10 percent and the best 10 percent of the distribution on each of the three dimensions were identified.

### Using relative scores or rankings

Relative rankings were used because we lack an absolute standard for most quality indicators. For example, what proportion of a home's residents could reasonably be expected to decline in ADL function over three months before considering the decline problematic? We really can't say. All we can say is that homes in which 20 percent of residents declined probably performed better than homes in which 40 percent of residents declined. The same statement holds true for state survey deficiencies. There is somewhat greater agreement on absolute staffing levels [21]. However, that agreement is not so substantial that the authors felt comfortable using those absolute measures. For the Quality Monitor, homes were ranked within each state. The percentile ranking within each state was used because staffing requirements, resident populations, and regulatory stringency vary by state.

The research team used percentile rankings. The use of percentile ranking introduces some distortion [6] into the measurement process (how different are the 89^th ^and the 91^st ^percentiles after all?) But, the authors believe that the face validity, intuitive appeal, and clarity for consumers of looking at the top 10 percent and the bottom 10 percent outweighed any concern about that distortion. We also believe our use of multiple measures minimizes the effect of this problem.

### Using longitudinal data

To determine a home's ranking on each dimension of quality, our analyses also used data from the three most recent surveys and the OSCAR reports accompanying the surveys. Previous research has shown the value of using longitudinal data [22]. Results from prior years were discounted, while the most recent data was given full value. The basic argument for this procedure is that the results for any single year, like those for any single indicator, can be anomalies. So, using multiple years or time periods is important. But, not all history is equally relevant. Those years or time periods closest to the present will be the best predictors of future behavior, so we weighted the results for the most recent time periods most heavily and results further removed in history the least heavily as we combined the results of the three most recent surveys.

### Looking at extremes

The validity of physical measurement is not usually dependent on the quantity being measured. A scale should give as accurate a weight for someone weighing 40 pounds, as for someone weighing 140 or 240. The same can rarely be said for social measurement. Any researcher who was analyzed the convergent validity of two scales in a cross-tabulation can testify to the differential congruence of the scales across the intensity of the stimuli. The disagreements between two scales (e.g., depression) or indicators (e.g., quality) are much more likely to occur in the middle-range of the scales than at either of the extremes.

Greater congruence or evidence of validity is found at the extremes of the scales. This argument is reinforced by some previous research that has shown that while absolute values for many quality indicators may be questionable, homes with relatively low ranking and homes with relative high rankings do differ in the expected direction in some areas of quality [23,24].

The measurement reality noted above and the difficulty of providing judgments about individual homes drove the authors, in the Quality Monitor, to look only at those facilities in the bottom or top 10 percent on each measure in each state (the extremes).

### Developing a single statement about a home

Recognizing all the problems with nursing home performance measurement is important. Nonetheless, we believe that we must make summary statements about homes. For regulators, policy-makers, and consumers we need to have a way of indicating whether Home A is, in general, better than Home B. Recognizing that the result for any single dimension of quality could be anomalous, we focused our attention on homes that were identified as providing poorer or better quality on at least two of our three dimensions (staffing, quality indicators and survey deficiencies. Those homes that rated poorly (worst ten percent) on at least two of our three dimensions of quality were identified as potentially poor quality homes. Homes that rated highly on at least two of the dimensions (best 10 percent) were identified as potentially better quality homes.

The term "potentially" is used with each assessment of a home. This is done because nursing home performance measurement is not a world of certainty and perfectly valid measures. Our best judgments in this area are simply likelihoods. We try to identify homes more likely to provide worse care than other homes, or homes more likely to provide better care than other homes.

### Asymmetry in nursing home performance measurement

The Quality Monitor identified homes that provided potentially very poor care and homes that provided potentially good care. But, should one have equal confidence in the judgments "at each end of the continuum?" We believe that judgments concerning poor quality are more likely to be useful than those identifying good quality homes. The reason for this judgment lies in the answers to two questions:

• If a home provides good care to the average resident, does that indicate that the home will meet the care requirements of other types of residents?

• If a home provides poor care to the average resident, does that indicate that the home will provide poor care to other specific types of residents?.

The diversity of nursing home residents is a major challenge to nursing home performance measurement. The authors believe that current measurement systems provide information about the needs and treatment of the "average" patient. But, no specific patient is average. All residents have special, individual needs. So, our ability to provide information about which homes are best for specific individuals is very limited. The homes identified as potentially very good may be good options. But, again, there is no certainty.

On the other hand, if a home fails to provide good care for the average resident, one has no logical reason to suspect that they have saved some sort of special caring skills for specific types of resident. These homes probably provide bad care to everyone who enters them. Again, no certainty exists. But none of us would suggest someone visit these homes on the off-chance that they provide good care for a specific type of resident.

## Summary

Nursing home performance measurement systems are an important advance. However, the most fundamental limitation of all nursing home performance measurement models is that none of them really allow one to hear "the residents' voices." Residents' and family members' perceptions of quality and judgments about how well care needs are being met do not come into this rather sterile world inhabited by prevalence rates, incidence rates, and percentile rankings. No analysis strategy will remedy this. Any solution must be built on new approaches to both quality measurement and quality assurance. The discussion of potential directions to go in search of these new approaches is beyond the scope of this work. Here the authors are attempting to minimize problems with the current approaches.

Another general limitation of all performance measurement strategies is that no matter what we believe or argue is logical or reasonable, we lack a gold standard to establish the validity of much of what we do. Unfortunately, no clear remedy is currently available for this problem. Logic and reason in combination with flawed data systems seem to be our only real refuge at this point.

While the silence of resident voices and the lack of a gold standard pervade all these quantitative models, the proposed strategy also has specific limitations not necessarily shared by other performance measurement strategies. A major limitation of this strategy is that it provides stakeholders information on only a limited subset of homes. The 2006 Quality Monitor contained only 1,008 nursing homes. Of these, 449 were classified as potentially poor homes, while 559 homes were identified as potentially good. The approximately 15,000 remaining facilities fell between our two extremes. Other strategies less stringent in their requirements (i.e., looking at the top and bottom quartiles) than the Quality Monitor algorithm, which emphasized the top and bottom 10% of homes, would have yielded a larger subset of homes. However, the authors believe that saying something about over 1,000 specific nursing homes that we believe have a relatively high likelihood of validity is superior to saying something about 5,000 nursing homes that may include greater error.

Despite its limitations, we present arguments for what is often considered an unusual thing. We suggest a step backwards. We argue that we can best serve policy-makers and consumers by eschewing nursing home reporting systems that provide information about all the facilities in a city, a state, or the nation on a website or in a report. We believe that our desire for a good performance measurement system for nursing homes (an undeniably admirable goal) has moved us to over-step the reach of our current data sources, data systems, and analytic abilities. We present what we believe to be a model of performance measurement more modest in its efforts ambitions and more useful in its results.

Nonetheless, even with our chosen strategy, which is extremely conservative, we sometimes failed to identify what seem to be bad homes. One of the nursing homes in New York State that was *not *classified as providing poor care in the Nursing Home Quality Monitor was recently the focus of an investigation by the Attorney General of New York State. Cameras placed in the home captured what the Attorney General alleges are graphic images of poor care, neglect, and abuse [25]. If these allegations are true, even our conservative approach to classification sometimes misses places where horrible things occur. Less modest approaches to nursing home performance measurement may do even worse.

## Competing interests

The author(s) declare that they have no competing interests.

## Authors' contributions

TL developed the original idea for the Watch List. MK worked with TL to develop the more comprehensive Quality Monitor. MP, CH, TL developed the specific structure for measuring quality. MK reviewed and provided comments on that structure. CP wrote the first draft of paper. TL, CH, and MK provided comments and proposed revisions. All authors have read and approved the final manuscript.

## Pre-publication history

The pre-publication history for this paper can be accessed here:


